# Acute adomen in a transplant patient with tuberculous colitis: a case report

**DOI:** 10.1186/1757-1626-2-9305

**Published:** 2009-12-10

**Authors:** Nikolaos Sikalias, Konstantinos Alexiou, Lamprini Mountzalia, Vasileios Triantafyllis, Georgios Efstathiou, Georgios Antsaklis

**Affiliations:** 1 Department of Surgery, "Sismanoglio" General Hospital of Athens, Sismanogliou 1, Marousi, Athens (15126), Greece; 2 Primary Health Care department, Health Center of Erythres, Erythres Ave 34, Erythres 32200, Greece

## Abstract

**Introduction:**

Tuberculous colitis is a rare form of tuberculosis and is found in immunosuppressed patients, usually with the clinical appearance of Crohn's disease. The purpose of this article is to report a rare case of tuberculous colitis in a transplant patient, presenting in the form of bowel obstruction and acute abdomen.

**Case presentation:**

**Methods**

A male patient, 51 years old, with a history of kidney transplant in a foreign country 19 months before, presented at the emergency department, after being referred by a primary care center, with obstipation during the previous week and acute abdominal pain. The patient had the clinical appearance of acute abdomen. Five days before the patient underwent colonoscopy, which revealed an intraluminal mass that partially occluded the lumen of the ascending colon. Blood tests revealed a mildly elevated WBC count and anaemia. Imaging studies revealed air in the peritoneal cavity and free fluid in the pelvis. The patient was diagnosed with perforation of a hollow viscus and was admitted for surgery

**Results**

During laparotomy the findings were a perforation of the cecum, a fragile mass (pseudopolyp), which occluded the lumen approximately in the middle of the ascending colon, diffuse erosions of the mucosa and pseudomembranes. A bezoar was found impacted at the level of the occlusion. There was also marked lymph node enlargement in the mesentery and ischaemia of the cecum. A typical right hemicolectomy was performed and special care was taken so, as not to damage the renal transplant. The pathological and microbiological (from free peritoneal fluid) investigation suggested the diagnosis of tuberculous colitis. Further blood tests and respiratory investigation confirmed the diagnosis of active tuberculosis, with the colon as a primary site. The patient received the appropriate antituberculous treatment.

**Conclusions**

Tuberculous colitis is a rare form of tuberculosis and is found in immunosuppressed patients. Deferential diagnosis from other forms of colitis is usually problematic. Tuberculous colitis rarely manifests itself in the form of acute surgical abdomen. The possible diagnosis of tuberculous colitis must be always in mind when treating transplant patients.

## Introduction

Tuberculosis of the colon is a very difficult clinical, diagnostic and therapeutic problem, as it is very hard to discover and even harder to prove if there is no clinical suspicion [1]. Sporadic cases of tuberculosis of the colon are reported in the international bibliography. This is due to the difficulty of identifying the *Mycobacterium tuberculosis *in the samples taken from lower GI endoscopy (identified in less than 18% of cases), as well as the fact that diffuse tuberculosis of the colon appears very similar with Crohn's disease [2,3]. Tuberculosis of the colon is rarer than pulmonary tuberculosis, with which it usually coexists, and it also appears to be more common in immunosuppressed patients [4,5]. Tuberculous colitis is a very rare form of tuberculosis found in immunosuppressed patients and its manifestation is usually similar to Crohn's disease.

## Case presentation

**Figure 1:**
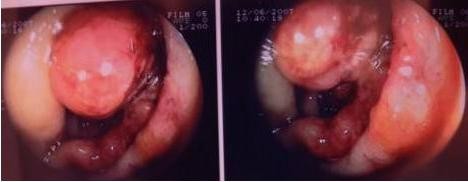
**Colonoscopic image of the patient**. Intraluminal mass, partially occluding the lumen of the ascending colon.

A Caucasian male Greek patient, 51 years old, with a history of kidney transplant in a foreign country 19 months before, presented at the emergency department, after being referred by a primary care center, with obstipation during the previous week and acute abdominal pain. The patient was receiving immunosuppressive medication (cyclosporine, MMF, prednisolone). The patient reported low fever (<37.5°C), which was attributed to cystitis, sweating, pain in the lower abdomen, anorexia, weakness and loss of weight during the previous 2 months. The patient also reported alternating constipation and non-haemorrhagic diarrhea in the previous 15 days. 5 days before, the patient underwent colonoscopy, which revealed an intraluminal mass, partially occluding the lumen of the ascending colon (figure 1).

Clinical examination revealed: fever (38.9°C), diffuse abdominal tenderness, especially at the right iliac fossa with intense signs of peritoneal irritation, no bowel sounds and marked tenesmus. Blood tests revealed a mildly elevated WBC (13.700/μl with 82.3% poly) and hypochromic microcytic anaemia. Imaging study revealed air in the peritoneal cavity and free fluid in the pelvis. The patient was diagnosed with hollow viscus perforation and was admitted for surgery.

During laparotomy the findings were a perforation of the cecum, a fragile mass (pseudopolyp), which occluded the lumen approximately in the middle of the ascending colon, diffuse erosions of the mucosa and pseudo membranes. A bezoar was found impacted at the level of the occlusion. There was also marked lymph node enlargement in the mesentery and ischaemia of the cecum. A typical right hemicolectomy was performed and special care was taken so, as not to damage the renal transplant. The pathological and microbiological (from free peritoneal fluid) investigation suggested the diagnosis of tuberculous colitis. Specifically, macroscopic study of the resected tissues revealed multiple ulcerations of the mucosa of the distal ileus, the cecum and the ascending colon. Microscopic examination revealed multiple submucosal granulomas, with aggregations of gigantic cells and localized necrosis of the granulomas, with rupture of the overlaying mucosa. Cryptic abscesses and diffuse inflammatory infiltration of the bowel mucosa, with concurrent thinning, were also observed. The image was compatible with chronic colitis. At the same time, multiple lymph node infiltrations of the mesentery and thrombosis of several distal vessels with local bowel ischaemia were found. *M. tuberculosis *was isolated from the material sent for culture.

From the blood and respiratory tests that followed, active tuberculosis was diagnosed, with the colon as the known primary site. During his stay at the hospital, the patient received broad spectrum antibiotics for the treatment of the fecal peritonitis and corticosteroids were discontinued. After the patient's exit, he received antituberculous treatment with 300 mg/24 h of isoniazide, 450 mg/24 h of rifampicine and 600 mg/24 h of ethambutole. The patient underwent two colonoscopies, 45 days and 3 months after his exit respectively, which were negative for *M. tuberculosis*.

## Discussion

The diagnosis of the tuberculosis of the colon can only be achieved if there is a high suspicion of the physician's part. It is based on the clinical appearance, endoscopy, blood and microbiological tests for the identification of the relevant microorganism. Tuberculosis of the colon accounts for 12.1% of the cases of GI tuberculosis and for 6% of the cases of intraabdominal primary location [4]. The histopatologic image form endoscopic biopsies are the primary criterion for the differential diagnosis from Crohn's disease [2,3,6].

As far as the clinical appearance is concerned, diarrhea is usually absent in the tuberculosis of the colon. Diarrhea is a constant criterion for the diagnosis of Crohn's disease [3,7], although in patients with very large intraluminal granulomas, which partially occlude the lumen, it can also appear in the tuberculosis of the colon. Diarrhea is also present in specific patients with overgrowth of the enteric flora with concurrent inflammation. Patients with tuberculous colitis or enteritis that have no pulmonary tuberculosis account for 19-50% [2,4]. Tuberculous colitis usually affects the distal ileus and the ileocecal region, which also complicates the differential diagnosis from Crohn's disease [1,3].

The histopatologic findings, either from colonoscopy or from colectomy of the affected colon, include the image of chronic colitis with ulceration of the mucosa, the growth of granulomas and lymph node infiltration. Pseudopolyps are also quite usual and if the grow near the ileocecal valve; they can cause disfigurement or even occlusion of the valve [3,6,8,9].

Granulomas with central necrosis can be found in tuberculous colitis as well as in Crohn's disease. In the case of small submucosal granulomas there is a serious differential diagnosis problem, if no microbiological tests are made. Large granulomatous pseudopolyps are a specific diagnostic criterion for tuberculosis. Hypertrophy of the colonic wall and the mesentery are usually found in both diseases. Thinning of the colonic wall and marked lymphadenopathy, with vascular ischaemia is more consistent with tuberculosis [9,10]. Even if tuberculous colitis is considered, a thorough microbiological work-up cannot always prove the disease. Stains and cultures for acid- fast bacilli in colon biopsies are positive in only 32-35% and 36-40% of patients respectively [5,9]. PCR (*M tuberculosis *DNA) from colonic biopsies is positive in 60% of patients [11] and has recently been favoured to differentiate intestinal Tuberculosis from Crohn's disease [12]. Imaging methods can provide some assistance when such clinical suspicion has been mentioned.

In the current bibliography many cases can be found, where it was impossible to have a definitive diagnosis, despite the exhaustive testing. In those cases antituberculous treatment was administered, when the treatment for Crohn's disease failed. In the cases where there is strong clinical suspicion and no definitive diagnosis, it is recommended to administer antituberculous treatment first, as it does not negatively affect Crohn's disease [13]. In every case where there is a suspicion of tuberculosis, it is highly recommended to avoid or discontinue corticosteroids, until every microbiological and serological test becomes negative. The treatment of tuberculous colitis can be achieved after 9-12 months of antituberculous treatment. This can be verified with the appropriate microbiological and serological tests, as well as the healing of the lesions, as seen with colonoscopy and biopsies [6,7,12].

## Conclusions

Tuberculous colitis is a rare form of tuberculosis and is found in immunosuppressed patients. It is difficult to differentiate from other types of colitis and it does not usually appear as acute abdomen. Tuberculous colitis must always be taken into account when treating transplant patients.

## Abbreviations

DNA: deoxyribonucleic acid; GI: tuberculosis; GT: gastrointestinal tuberculosis; MMF: Mycophenolate Mofetil; PCR: Polymerase Chain Reaction; TB: Tuberculosis.

## Consent

Written informed consent was obtained from the patient for publication of this case report and accompanying images. A copy of the written consent is available for review by the journal's Editor-in-Chief.

## Competing interests

The authors declare that they have no competing interests.

## Authors' contributions

SN, AK, TV, AG have had an equally substantial contribution to the clinical diagnosis, surgical management and post-op follow-up of the patient. EG and SN drafted the manuscript. ML is the Referring Doctor. SN and AG are guarantors of the paper. All authors read and approved the final manuscript.
